# Expression and prognostic significance of cancer-testis antigens (CTA) in intrahepatic cholagiocarcinoma

**DOI:** 10.1186/1756-9966-30-2

**Published:** 2011-01-06

**Authors:** Jin-xue Zhou, Yin Li, Sun-xiao Chen, An-mei Deng

**Affiliations:** 1 Department of Hepatobiliary and Pancreatic Surgery, Henan Tumor Hospital, Zhengzhou, Henan 450008, PR China; 2 Capital Medical University School of Stomatology, Beijing 100050, PR China; 3 Changzheng Hospital, Second Military Medical University, Shanghai 200003, PR China

## Abstract

**Background:**

Cancer-testis antigens (CTAs) are suitable targets for cancer-specific immunotherapy. The aim of the study is to investigate the expression of CTAs in intrahepatic cholagiocarcinoma (IHCC) and evaluate their potential therapeutic values.

**Methods:**

Eighty-nine IHCC patients were retrospectively assessed for their expression of CTAs and HLA Class I by immunohistochemistry using the following antibodies: MA454 recognizing MAGE-A1, 57B recognizing multiple MAGE-A (MAGE-A3/A4), E978 recognizing NY-ESO-1, and EMR8-5 recognizing HLA class I. The clinicopathological and prognostic significance of individual CTA markers and their combination were further evaluated.

**Results:**

The expression rates of MAGE-A1, MAGE-A3/4 and NY-ESO-1 were 29.2%, 27.0% and 22.5%, respectively. The concomitant expression of CTAs and HLA class I antigen was observed in 33.7% of the IHCC tumors. We found that positive MAGE-3/4 expression correlated with larger tumor size (≥ 5 cm), tumor recurrence and poor prognosis. Moreover, we identified 52 cases (58.4%) of IHCC patients with at least one CTA marker expression, and this subgroup displayed a higher frequency of larger tumor size and a shorter survival than the other cases. Furthermore, expression of at least one CTA marker was also an independent prognostic factor in patients with IHCC.

**Conclusion:**

Our data suggest that specific immunotherapy targeted CTAs might be a novel treatment option for IHCC patients.

## Introduction

Intrahepatic cholagiocarcinoma (IHCC) is a relatively uncommon malignancy, comprising approximately 5%-10% of the liver cancers, and both its incidence and mortality have increased in recent years in China and other countries [1,2]. IHCC is not sensitive to radiation therapy and chemotherapy. Even the patients undergoing a radical surgical resection is still at a high risk for early recurrence, and the patients' survival is thus unsatisfactory. Therefore, there is a great need to identify molecular targets for developing novel therapeutic approaches for patients with IHCC.

Cancer testis antigens (CTAs) comprise a group of non-mutated self-antigens selectively expressed in various tumors and normal testis tissues, but not in other normal tissues [3]. Several studies have shown that if presented with human leukocyte antigen (HLA) class I molecules, these tumor-associated antigens could induce effective anti-tumor cytotoxic T lymphocytes (CTLs) response in vitro and in vivo [4]. Because of these unique characteristics, CTAs are regarded as promising targets for cancer-specific immunotherapy [5]. However, the possibility that IHCC patients might benefit from CTA-targeted therapies has not been evaluated.

Given their potential therapeutic significance, it may have significance for exploring the presence of CTAs in IHCC. However, to our knowledge, until now, only two studies examined the mRNA and protein expression of CTAs in small number of IHCC cases [6,7]. The CTAs expression at protein level and their clinicopathological and prognostic significance in a larger cohort have not been investigated.

The aims of the current study were to analyze the expression of MAGE-A1, MAGE-A3/4 and NY-ESO-1 CTAs in IHCC tissues by immunohistochemistry, and to investigate correlations between their expression with HLA class I expression, clinicopathologic parameters and survival in patients with IHCC.

## Materials and methods

### Patients

The study was approved by the research ethics committee of our institutions, and informed consent was obtained from each patient. A total of consecutive 102 patients with IHCC who underwent curative resection at Department of Hepatobiliary and Pancreatic Surgery, Henan Tumor Hospital (Zhengzhou, China) and Changzheng Hospital (Shanghai, China) from 1999 to 2006 were retrospectively reviewed. Patients with lymphnode-positive metastasis routinely received 5-fluorouracil-based chemotherapy, and Gemcitabone chemotherapy was given when recurrence occurred. Patients were followed up every two month during the first postoperative year and at every four month afterward. Follow-up was finished on May 2008. The median follow-up was 24 month (range, 4-61 month). Overall survival (OS) time was defined as the time from operation to cancer-related death only.

Cases were included according to the following inclusion criteria: having archived formalin-fixed, paraffin-embedded specimens available; having complete clinicopathological and followed-up data; receiving no anticancer treatment before operation. Patients who died of unrelated diseases and within one month after operation were excluded, leaving 89 patients eligible for this analysis. The clinical and pathological details of these patients were summarized in Additional file 1.

### Immunohistochemical analysis

Immunohistochemical analysis was performed on archived tissue blocks containing a representative fraction of the tumors. Briefly, 5-μm-thick paraffin-embedded tissue sections were deparaffinized and rehydrated. Endogenous peroxidase was blocked with methanol and 0.3% H_2_O_2 _for 20 min. Antigen retrieval was performed with microwave treatment in 0.1 M sodium citrate buffer (pH 6.0) for 10 min. Expression of CTAs was detected with the monoclonal antibody against MAGE-A1 (clone MA454), MAGE-A3/4 (clone 57B) and NY-ESO-1 (clone E978), as described previously [8-10]. Clone 57B was originally raised against MAGE-A3, and later has been reported to primarily recognize the MAGE-A4 antigen [11,12]. Currently, 57B is considered to be anti-pan-MAGE-A (MAGE-A3/4). Expression of HLA class I was detected with an anti-pan HLA class I monoclonal antibody EMR8-5, as described previously [13]. Detection was performed with the Dako Envision system using diaminobenzidine (DAB) as the chromogen. Non-specific mouse IgG was used as negative control and normal human testis tissues were used as positive controls for CTA expression. Immunochemical results were evaluated and scored by two and independent observers according to the previous criteria [14]. Positive CTA expression was assigned to any extent of immunostaining in sections and further graded into four groups: + : < 5% of tumor cells stained; ++ : 5-25% of tumor cells stained; +++ : > 25-50% of tumor cells stained; ++++ : > 50% of tumor cells stained. A patient was considered CTA-positive if at least one of three markers demonstrated positive immunoactivity. HLA class I expression was classified as positive and down-regulated compared with stromal lymphocytes as an internal control as previously described [13].

### Statistical analysis

The associations between CTAs expression and clinicopathological parameters were evaluated using Chi-square or Fisher's exact test, as appropriate. Overall survival of patients were estimated by the Kaplan-Meier method, differences between groups were compared were by the log-rank test. Multivariate analysis was performed using a Cox proportional hazard model. Statistically significant prognostic factors identified by univariate analysis were entered in the multivariate analysis. All the statistical analyses were performed with SPSS 16.0 software. P value less than or equal to 0.05 was considered statistically significant.

## Results

### Expression of MAGE-A1, MAGE-A3/4, NY-ESO-1 and HLA class I proteins in IHCC patients by immunohistochemistry

**Figure 1:**
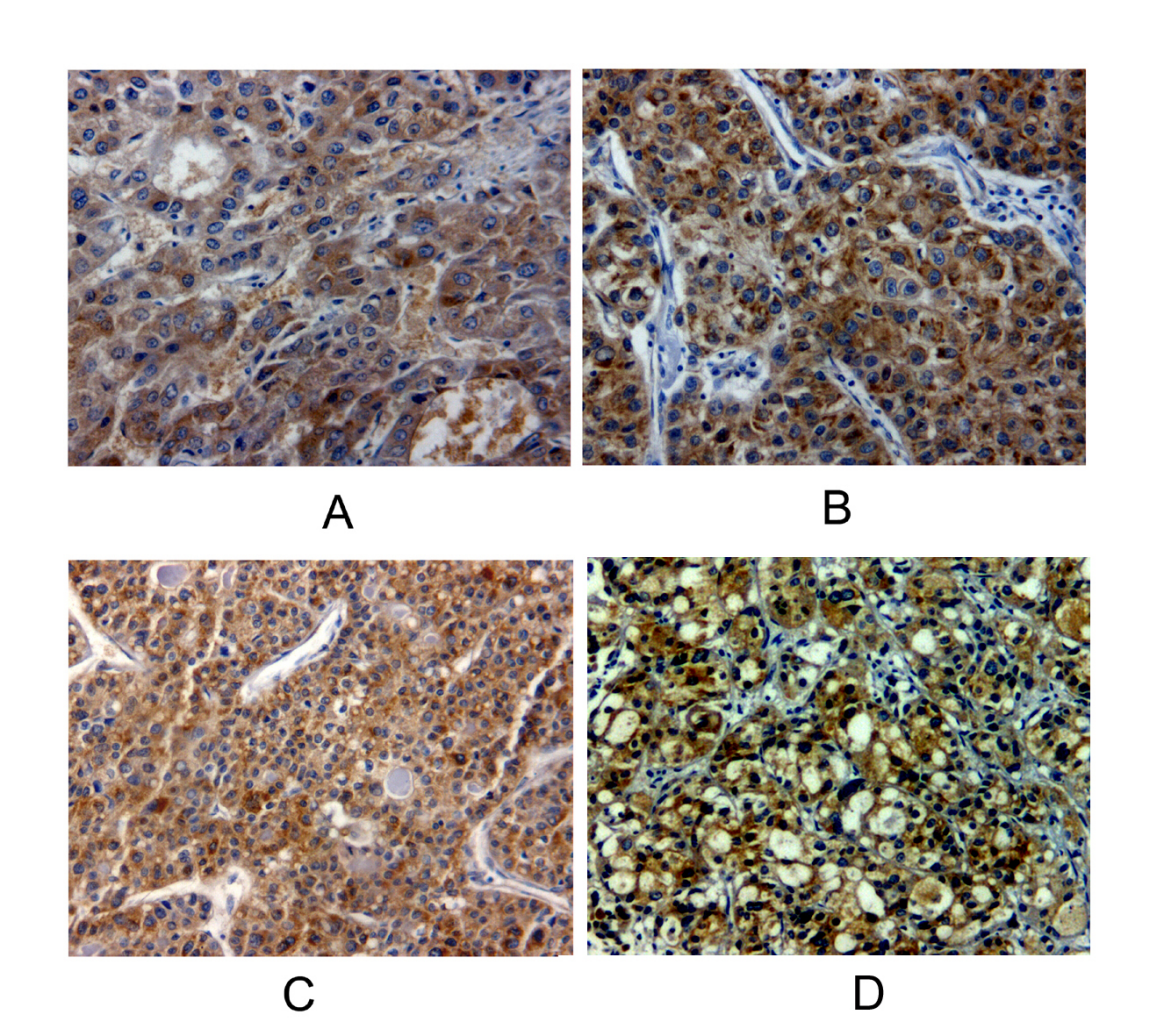
**Immunohistochemical analysis of MAGE-A1, MAGEA3/4, NY-ESO-1 and HLA Class I in intrahepatic cholagiocarcinoma**. Sections were stained with antibody against (A) MAGE-A1 (MA454); (B) MAGE-A3/A4 (57B); (C) NY-ESO-1 (E978); (D) HLA Class I (EMR8-5).

**Figure 2:**
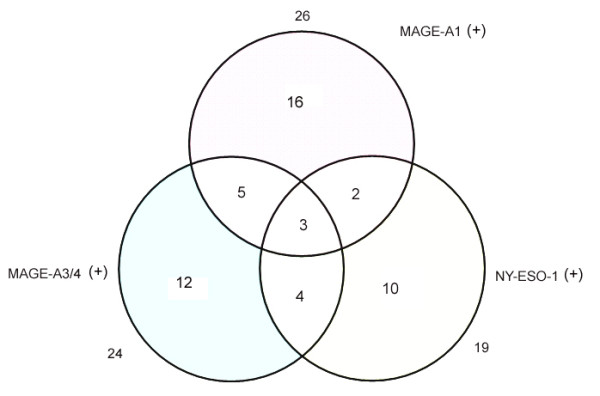
**Venn diagram depicting the overlap in the expression of cancer-testis antigens in intrahepatic cholagiocarcinoma**.

MAGE-A1, MAGE-A3/4 and NY-ESO-1 showed a predominantly, although not exclusively, cytoplasmic staining (Figure 1). The frequency and grade of various CTA expressions in tumors is shown in Table 1. Figure 2 showed a Venn diagram dipicting the overlap of three CTAs expression. When the CTA combinations were tested, 52 from 89 IHCC cases (58.4%) showed expression of at least one marker, 14 cases (15.7%) demonstrated co-expression of two CTAs, and only three cases (3.3%) were positive for all the three antigens. As seen in table 2, down-regulated HLA class I expression was found in 42.7% of all tumors (n = 38). Comparing the relationship between individual or combined CTAs expression and HLA-class I expression, no correlation was observed. And 30 IHCC cases (33.7%) demonstrated concomitant expression of CTAs and HLA class I antigen.

**Table 1:** Expression of cancer-testis antigens in intrahepatic cholanglocarcinoma

	MAGE-A1 N (%)	MAGE-A3/4 N (%)	NY-ESO-1 N (%)
**Negative**	63 (70.8)	65 (73.0)	70 (78.7)
**Positive**	26 (29.2)	24 (27.1)	19 (21.3)
+	2 (2.2)	1 (1.1)	1 (1.1)
++	3 (3.4)	4 (4.4)	1 (1.1)
+++	12 (13.5)	14 (15.7)	7 (7.9)
++++	9 (10.1)	5 (5.6)	10 (11.2)

**Table 2:** Correlation between CTA expression pattern and HLA class I expression

CTA expression pattern	HLA class I expression		P value
		
	Positive (n = 51)	Down-regulated (n = 38)	
**MAGE-A1**			
Positive	18	8	0.144
Negative	33	30	
**MAGE-A3/4**			
Positive	11	13	0.184
Negative	40	25	
**NY-ESO-1**			
Positive	11	8	0.953
Negative	40	30	
**1 CTA positive**			
With	30	22	0.930
Without	21	16	
**2 CTA positive**			
With	9	5	0.565
Without	42	33	
**3 CTA positive**			
With	1	2	0.795
Without	50	36	

### Correlation between CTAs expression with HLA-class I expression and clinicopathological parameters

We found that positive MAGE-A3/4 and one CTA marker expression were detected more frequently in tumors with bigger size (≥ 5 cm) (20/24, 38/52), than in smaller tumors (P = 0.011, P = 0.009). In addition, MAGE-A3/4 positive IHCC had a higher recurrence rate (17/24) than negative subgroup (30/65, P = 0.038). There was no statistically significant correlation found between individual or combined CTA expression and any other clinicopathological traits.

### Correlation between CTAs expression and overall survival

The correlation of clinicopathological parameters and individual or combined CTA expression with overall survival was further investigated. As shown in Table 3, univariate analysis showed that overall survival significantly correlated with TNM stage, lymphnode metastasis, resection margin, differentiation and tumor recurrence but not with gender, age, tumor size and number, vascular invasion and perineural invasion.

**Table 3:** Univariate analyses of prognostic factors associated with overall survival (OS)

Variable	Category	No. of patients	P
Gender	female vs. male	31 vs. 58	0.587
Age	< 60 vs. ≥60, years	19 vs. 70	0.532
TNM stage	1/2 vs. 3/4	34 vs. 55	0.007
Tumor size	≥5 cm vs. < 5 cm	55 vs. 34	0.690
Differentiation	well or mod vs. poor	26 vs. 63	0.008
Resection margin	R0 vs. R1/2	56 vs. 33	0.008
Tumor number	single vs. multiple	58 vs. 31	0.385
Vascular invasion	with vs. without	42 vs. 47	0.227
Perineural invasion	with vs. without	33 vs. 56	0.736
Lymph node metastasis	with vs. without	38 vs. 51	0.001
Tumor recurrence	with vs. without	47 vs. 42	0.022
MAGE-A1	Positive vs. negative	26 vs. 63	0.116
MAGE-A3/4	Positive vs. negative	24 vs. 65	0.009
NY-ESO-1	Positive vs. negative	19 vs. 70	0.068
1 CTA positive	with vs. without	52 vs. 37	0.001
2 CTA positive	with vs. without	14 vs. 75	0.078
3 CTA positive	with vs. without	3 vs. 86	0.372

**Figure 3:**
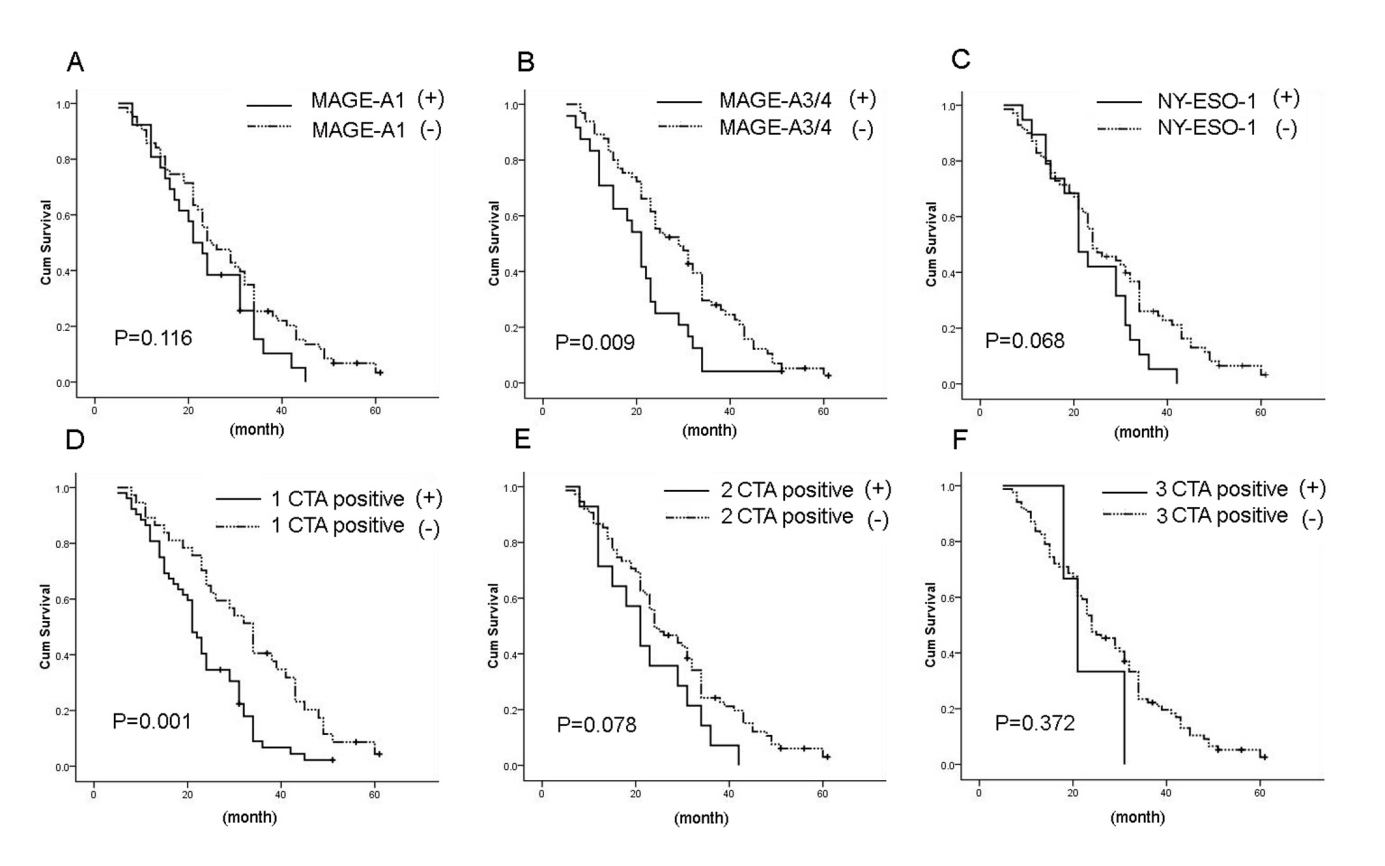
**Correlation between individual or combined CTA expression and survival**. Kaplan-Meier survival curves performed according to CTAs expression.(A) MAGE-A1; (B) MAGE-A3/4; (C) NY-ESO-1; (D) at least one CTA positive; (E) two CTAs expression; (F) with three CTAs expression.

Patients with MAGE-A3/4 positive tumors had a significantly poorer outcome compared to those without MAGE-A3/4 expression. MAGE-A1 and NY-ESO-1 also demonstrated the same trend but did not reach statistical significance. Interestingly, negative expression in all CTAs correlated with a better prognosis than at least one CTAs expression, meanwhile, two or three CTAs expression had no impact on survival (Figure 3, Table 3). COX proportional hazard model analysis showed that at least one CTA expression was an independent prognostic indicator for IHCC, whereas the association of MAGE-A3/4 with a shorter survival failed to persist in the multivariate analysis (Table 4).

**Table 4:** Multivariate analyses of factors associated with overall survival (OS)

Variable	HR	95% Confidence Interval		P value
			
		Lower	Upper	
1 CTA positive	0.524	0.298	0.920	0.024
MAGE-A3/4	0.897	0.505	1.594	0.711
Differentiation	0.447	0.263	0.758	0.003
TNM stage	1.122	0.597	2.110	0.721
Lymph node metastasis	0.389	0.207	0.732	0.003
Tumor recurrence	0.706	0.386	1.291	0.258
Resection margin	1.138	0.574	2.258	0.711

## Discussion

In this study, expression of three CTAs at protein level was investigated by immunohistochemistry. MAGE-A1, MAGE-A3/4 and NY-ESO-1 were selected considering that these antigens have been well-accredited and are being applied for clinical trials of vaccine immunotherapy [15-18]. The expression frequency of CTAs varies greatly in different tumors type [19,20]. Our results showed that expression rates of MAGE-A1, MAGE-A3/4 and NY-ESO-1 in IHCC were less than 30%. According to the established criteria [21], IHCC should be classified to be low "CTA expressors". In a previous study, the expression rates of MAGE-A1, MAGE-A3 and NY-ESO-I in IHCC were 20.0% (4/20), 20.0% (4/20) and 10.0% (2/20) detected by RT-PCR [6]. However, in the immunohistochemical study by Tsuneyama et al. [7], 32 of 68 IHCC cases (47.1%) demonstrated positive MAGE-A3 expression using a polyclonal antibody. These discrepancies between our and previous studies may be related to the difference in the method of detection, the antibodies adopted and patient populations.

In this study, we also identified that only MAGE-3/4 and at least one positive CTA expression correlated aggressive phenotypes including bigger tumor size and higher recurrence rate. There was no other association observed between CTA markers (either individual or combined) with HLA class I expression and clinicopathological parameters of IHCC patients.

Curves of patients with positive for the individual or multiple CTAs (with two or three CTA positive) markers leaned towards a poorer outcome, however, only MAGE-A3/4 reach statistical significance. We speculated that such statistically insignificant trends were likely to be due to the fact that only a small number of IHCC cases presented with positive CTA expression (either individual or co-expressed) in this study. Considering that combination of CTAs makers may reinforce the predictive value for prognosis and malignant phonotype by one single CTA alone, we next asked whether at least one CTA expression had n significant impact on outcome. We found that at least one CTA expression did indeed correlate with a significantly poorer survival. Furthermore, at least one positive CTA expression was also an independent prognostic factor for patients with IHCC.

Interestingly, in this study, MAGE-A1 and NY-ESO-1 positive IHCC tumors seem to have a relatively higher frequency of positive expression of HLA class I than MAGE-A3/4 positive cases. Recently, Kikuchi et al. [22] indicated that co-expression of CTA (XAGE-1b) and HLA class I expression may elicit a CD8+ T-cell response against minimal residual disease after surgery and resulted in prolonged survival of NSCLC patients, while expression of CTA combined with down-regulated HLA class I expression correlated with poor survival. Therefore, we speculated that a relatively high proportion of HLA Class I-negative cases in MAGE-A3/4 positive group may partly account for its association with significantly poor survival.

MAGE-A1, MAGE-A3/4 and NY-ESO-1 have been applied for clinical trials of vaccine immunotherapy for multiple cancer patients, but the utility of CTA immunotherapy against patients with IHCC remains investigated. In this study, using three CTA markers MAGE-A1, MAGE-A3/4 and NY-ESO-1, we identified a subgroup (58.4%) of IHCC patients with at least one CTA expression having a poor prognosis. Moreover, high levels of expression of these antigens were observed in most positive cases. In our study, the concomitant expression of CTAs and HLA class I antigen was observed in 33.7% of the IHCC tumors, which indicating that it may be possible to immunise a significant proportion of IHCC patients with tumor-specific CTLs. Based on our data, we suggest that a considerable number of IHCC patients at high-risk might benefit from specific immunotherapy targeted MAGE-A and NY-ESO-1.

This is the first study demonstrating a correlation between CTA and prognosis in IHCC. Furthermore, this present retrospective cohort study is limited to relatively small case series (although more than previous studies); therefore, further validation will be required before these antigens can be tested for targeted immunotherapy.

## Conclusion

In conclusion, our data suggest that the cancer-testis antigens identified in this study might be novel biomarkers and therapeutic targets for patients with IHCC.

## Competing interests

The authors declare that they have no competing interests.

## Authors' contributions

JXZ and YL contributed to clinical data, samples collection, immunohistochemistry analysis and manuscript writing. SXC and AMD were responsible for the study design and manuscript writing. All authors read and approved the final manuscript.

## Supplementary Material

Additional file 1 **Table S1 Clinicopathological characteristics of patients included in this study**. a table for the clinicaopathological characteristics of 89 IHCC patients. Click here for file
