# Supplementary material for: Expression and prognostic significance of cancer-testis antigens (CTA) in intrahepatic cholagiocarcinoma
Source: J Exp Clin Cancer Res. 2011 Jan 6;30(1):2. doi: 10.1186/1756-9966-30-2 (PMC3023685; doi:10.1186/1756-9966-30-2)
Supplement: Additional file 1 — Table S1 Clinicopathological characteristics of patients included in this study. a table for the clinicaopathological characteristics of 89 IHCC patients. [file 1756-9966-30-2-S1.DOC]

**Table S1. Clinicopathological characteristics of patients included in this study**

| **Variable** | **Category** | **Number** |
| --- | --- | --- |
|
| Gebder | male | 58 |
|  | female | 31 |
| Age (y) | <60 | 70 |
|  | ≥60 | 19 |
| TNM stage | 1/2 | 34 |
|  | 3/4 | 55 |
| Tumor size (cm) | <5 cm | 34 |
|  | ≥5 cm | 55 |
| Differentiation | well or mod | 26 |
|  | poor | 63 |
| Resection margin | R0 | 56 |
|  | R1/2 | 33 |
| Tumor number | single | 58 |
|  | multiple | 31 |
| Vascular invasion | yes | 42 |
|  | no | 47 |
| Perineural invasion | yes | 33 |
|  | no | 56 |
| Lymph node metastasis | yes | 38 |
|  | no | 51 |
| Tumor recurrence | yes | 47 |
|  | no | 42 |
